# Expanding access to sexual and reproductive health and rights through evidence-based policy dialogue: implications for practice from a multicountry initiative

**DOI:** 10.1136/bmjgh-2024-016587

**Published:** 2025-04-02

**Authors:** Ulrika Rehnstrom Loi, Amy Coates, Offeibea Obubah, Frank Noij, Katy Footman, Antonella Lavelanet, Hyobum Jang, Laurence Codjia, Laurence Läser, Tesfaye Tufa, Leopold Ouedraogo, Nilmini Hemachandra, Karima Gholbzouri, Meera Upadhyay, Neena Raina, Dina Gbenou, Souleymane Zan, Thierry Tossou Boco, Theopista John Kabuteni, Maria Mugabo Mujawamariya, Priya Karna, Ram Chahar, Amrita Kansal, Ellen Thom, Qudsia Uzma, Dan Kass, Carisse Hamlet, Adam Karpati, Shambhu Acharya, Bela Ganatra

**Affiliations:** 1 UNDP/UNFPA/UNICEF/WHO/ World Bank Special Programme of Research, Development and Research Training in Human Reproduction (HRP), World Health Organization, Geneva, Switzerland; 2 Department of Country Strategy and Support, World Health Organization, Geneva, Switzerland; 3 Department of Health Workforce, World Health Organization, Geneva, Switzerland; 4 World Health Organization Regional Office for Africa, Brazzaville, Congo; 5 World Health Organisation Regional Office for the Eastern Mediterranean, Cairo, Egypt; 6 World Health Organization Regional Office for South-East Asia, New Delhi, India; 7 World Health Organization, Ouagadougou, Burkina Faso; 8 World Health Organization, Cotonou, Benin; 9 World Health Organization, Kigali, Rwanda; 10 World Health Organization, New Delhi, India; 11 World Health Organization, Kathmandu, Nepal; 12 World Health Organization, Islamabad, Pakistan; 13 Vital Strategies, New York, New York, USA

**Keywords:** Health policy, Health systems

## Abstract

**Introduction:**

Policy dialogue is an important component of evidence-based policy-making. In 2019, WHO and Ministry of Health staff in 15 countries participated in an initiative that involved training and implementation of country-level sexual and reproductive health and rights (SRHR) policy dialogues. An evaluation of the process and outcomes was subsequently undertaken in six of the countries.

**Methods:**

The three-stage policy dialogue initiative included a preparatory phase to develop stakeholder analyses and policy briefs and a 2-day workshop to develop an action plan, followed by continuous support as the national teams implemented the action plans. A participatory, multimethod approach was used to evaluate the policy dialogue initiative, including a desk review of initiative documentation and interviews with project participants.

**Results:**

Participants reported positive experiences of the policy dialogue initiative and felt it improved their knowledge, skills and confidence. The ensuing policy dialogue activities in each country contributed to some SRHR policy development and/or implementation changes. The policy dialogue initiative supported these changes through its practical approach to learning and ongoing technical support. However, the impact of policy dialogues varied depending on political factors, the scope of policy goals, alignment with existing country priorities and stakeholder engagement. Furthermore, facilitating factors included strong support networks, incremental working and preparation for backlash against SRHR.

**Conclusions:**

Our experience highlights the value of policy dialogue for progressing SRHR policy change at the national level and the need for further investments in strengthening the skills of health decision-makers required for effective policy dialogue.

**WHAT IS ALREADY KNOWN ON THIS TOPIC**

- Policy dialogue is an important component of evidence-based policy-making; nonetheless, the process of policy dialogue and its effectiveness is rarely studied, particularly for sexual and reproductive health and rights (SRHR).

**WHAT THIS STUDY ADDS**

- Directed SRHR policy dialogue initiatives can support policy adoption and implementation; however, several enabling factors are required for effective policy dialogue in SRHR.

**HOW THIS STUDY MIGHT AFFECT RESEARCH, PRACTICE OR POLICY**

- The research indicates the need for continued investment in and support for SRHR policy dialogue and highlights factors that can support the integration of SRHR into broader health policy dialogues.

## Introduction

In much of the world, sexual and reproductive health and rights (SRHR) are not fulfilled and people lack sufficient access to a full range of SRHR services due to a range of legal and policy barriers.^1^ Policy dialogue is an important component of policy-making processes that can support priority setting, consensus-building and collective leadership.^2 3^ Although policy dialogue is not well defined, it is broadly understood as an iterative and deliberative process where evidence-based stakeholder discussions are used to develop a plan, strategy or policy for a targeted problem.^4 5^ Through enhanced quality, transparency and breadth of engagement with stakeholders, policy dialogue can increase trust and ownership of new policies, supporting implementation.^5^ Policy dialogues may address a common institutional challenge in Ministries of Health (MoHs)—that positions of authority are often occupied by well-trained technical personnel with technical, programme and managerial experience, but less so by personnel specifically able to manoeuvre the challenges of policy formation, advocacy and political approval.^6 7^ Policy dialogues are particularly important for pursuing universal health coverage (UHC), which requires collaboration from many actors.^2^ Although research has explored perceptions of policy dialogue in low-income and middle-income countries, the process of policy dialogue has rarely been documented.^4^ Despite considerable investment in advocacy for family planning,^8^,^11^ challenges with evaluating the impact or effectiveness of advocacy initiatives^12^ make it all the more important for these activities to be carefully documented and analysed.^13^

The WHO’s 13th general programme of work for 2019–2023 included a focus on policy dialogue, tailored to the country’s needs and context.^14^ In 2019, an SRHR policy dialogue initiative was implemented by the Human Reproduction Programme (the UNDP/UNFPA/UNICEF/WHO/World Bank Special Programme of Research, Development and Research Training in Human Reproduction) and WHO. The initiative aimed to build capacity and provide support for national SRHR policy dialogue implementation among participating countries. In this paper, we describe the policy dialogue initiative and share insights and lessons learnt from its evaluation.

## Methods

### The policy dialogue intervention

The policy dialogue intervention encompassed a variety of learning methods and approaches. Participants engaged in lectures, facilitated discussions, expert feedback sessions and interactive activities aimed at enhancing their understanding and application of policy processes, stakeholder engagement and effective communication techniques.

The initiative, delivered for MoH and WHO staff in 15 countries and evaluated in 6 (table 1), was designed and delivered by HRP and WHO in collaboration with Vital Strategies, a US-based global public health organisation specialising in public health system capacity building. The criteria for country selection were based on demographic data, interest in accelerating access to SRHR and donor priorities. Staff from 15 countries completed the initiative in two cohorts (table 1).

**Table 1:** Countries that participated in the policy dialogue initiative

Cohort 1		Cohort 2	
Country	Number of staff	Country	Number of staff
Benin*	3	Egypt	2
Burkina Faso*	2	Kenya	2
India*	3	Liberia	1
Nepal*	2	Malawi	3
Pakistan*	3	Mali	3
Rwanda*	3	Nigeria	3
South Africa	3	Sierra Leone	2
		Uganda	3

* Included in the evaluation.

The initiative was organised in three stages. In the first 3 month-preparatory stage, country teams comprised at least one technical staff member from the WHO country office and MoH (hereafter referred to as ‘national teams’). Capacity building and preparatory work began with support from technical experts from WHO’s Regional Offices and Headquarters and from Vital Strategies. Vital Strategies conducted webinars to orient each team to the policy dialogue process including data analysis, policy development and advocacy. Participants were encouraged to select an area of SRHR policy that addressed a major national concern and priority and responded to a policy gap. For each of the selected policy areas (table 2), national teams developed a draft policy brief and stakeholder analysis. Vital strategies provided a standardised template and quality criteria for policy briefs to country offices at this stage.

**Table 2:** Policy areas and progress in each country

Country	Policy area	Policy changes*
Benin	Inclusion of comprehensive abortion care in health insurance mechanisms	Health insurance benefits package piloted in selected health zones and evaluated for scale-up at the national level. Additional recommendations were made to include abortion and other sexual and reproductive health services in the health benefit package, which are now under discussion for inclusion.
Burkina Faso	Integration of family planning services into primary healthcare	MoH plan developed for integration of all family planning methods in all levels of health facility. Pilot project implemented in one area to evaluate benefits and inform a national-level plan. All family planning methods integrated into all levels of health facilities across the majority of districts, supported by MoH and partner organisations.
India	Inclusion of two SRHR indicators (relating to family planning and adolescent reproductive health) in the Government’s Transformation of Aspirational Districts Programme, a high-profile programme focused on social development	Two reproductive health indicators (adolescent fertility rate and modern contraceptive prevalence rate) were included in the Transformation of Aspirational Districts Programme in November 2019. Medical Termination of Pregnancy Act was amended to raise the gestational limit for abortion in 2021.
Nepal	Decentralisation and simplification of certification for safe abortion facilities	Policy for certification of safe abortion facilities accepted at the provincial level through order of the Ministry. Certification initiative implemented, enabled by new government funding. Provincial committees established to select and validate sites for certification (136 in total). Service providers underwent capacity training (300 in 2021). Decentralisation of abortion facility certification included in comprehensive abortion care guidelines.
Pakistan	Implementation of mandatory notification systems for maternal and perinatal death surveillance and response (MPDSR), including deaths from unsafe abortion	Draft bills for mandatory MPDSR had consultative meetings in each province and are awaiting review by respective health departments before submission to parliament. MPDSR system support incorporated into the country’s new UN Sustainable Development Framework for 2023–2027. Safe abortion is included in the essential package of health services.
Rwanda	Integration of SRHR (family planning, maternity services and postabortion care) into the essential package of health services	WHO guidelines for comprehensive abortion care, family planning and adolescent SRH adopted. Essential medicines list revised to include mifepristone, misoprostol and the combi-pack.

* These are based on changes to policy reported at the point of evaluation (2022) but have been updated to reflect additional policy changes achieved since the evaluation was completed.

MoH, Ministries of Health; SRHR, sexual and reproductive health and rights.

The second stage comprised a 2-day workshop in Addis Ababa, Ethiopia in September 2019 in which national teams refined their policy briefs and stakeholder analyses and developed an action plan for conducting policy dialogue towards national adoption. The workshop involved mentored working sessions for the national teams followed by group sessions, presentations and discussions where all participants provided peer review and feedback on other national teams’ work. These sessions covered: problem clarification, root-cause analysis, comparative policy benefits analysis, how to address common challenges with policy brief development, how to engage MoH and the role of WHO. The initiative also encouraged participants to have an ‘elevator pitch’ prepared, with supporting data and documents, which enabled teams to seek opportunities for policy dialogue activities. Professional media training was also incorporated into the agenda. The workshop design promoted hands-on learning from policy dialogue experts and peer-to-peer networking to facilitate ongoing support and problem-solving.

In the third stage, national teams implemented country-level policy dialogue action plans with dedicated support from all three levels of the WHO, HRP and Vital Strategies for 6 months. Group webinars, coaching check-in calls and on-demand technical support were provided to country teams. During this stage, policy dialogue activities implemented by national teams included numerous formal and informal meetings with government officials in the MoH and other sectors, establishment and coordination of technical advisory groups and stakeholder engagement. Policy briefs developed in earlier stages were used to guide discussions during these meetings and engagements. In some countries, policy dialogue activities were supported by additional evidence generation/data collection activities to further demonstrate and engage stakeholders in discussions concerning the need for the proposed policy, its feasibility and its benefits in the local context. In some countries, policy dialogue activities were supplemented by financial and technical implementation support for the proposed policy change.

### Evaluation of the policy dialogue initiative

In November 2022, an independent evaluator (FN) was appointed to design and conduct a rapid evaluation of the initiative with the objective to document, analyse and synthesise the achievements and good practices developed as a result of the project and to identify challenges and lessons learnt. The evaluation assessed changes in participant knowledge and competency, policy outcomes and the process by which policy achievements were attained.

Six of the 15 country teams engaged in the initiative participated in the evaluation. These countries were strategically important for the overall goals of the initiative and were purposefully selected to ensure geographical representation, availability of reliable data and access to relevant stakeholders.

A participatory, multimethod approach was used, triangulating evidence from multiple sources. This included a desk review of initiative documentation, such as donor reports, postworkshop feedback surveys and a quality assessment of the policy briefs and stakeholder analyses. The evaluation also included virtual semistructured interviews with project participants.

The independent evaluator conducted a quality assessment of the policy briefs and stakeholder analyses using a standardised framework to give each document a score from 0 to 10 based on adherence to the best practice guidelines for development used in training (0=lowest adherence/quality and 10=highest adherence/ quality) (table 3).

**Table 3:** Quality assessment of policy briefs

Country	Clearly written with summary	SRHR-related problem identified	Clear health outcome provided	Context and stake-holder analysis	Text and data visualisation	Selection among alternative solutions	Action steps and time frame	Overall assessment
Benin	Low	High	Low	Low	Low	Lowest	High	Low
Burkina Faso	Low	Highest	High	Low	Low	Lowest	Low	Low
India	Highest	Highest	High	Low	Highest	Lowest	High	High
Nepal	High	High	Low	Low	High	Lowest	High	High
Pakistan	High	Highest	High	Low	High	Low	Low	High
Rwanda	High	High	Lowest	High	Low	Lowest	Low	Low

Ratings lowest: 0.0–2.4, Low: 2.5–4.9 High: 5.0–7.4, Highest: 7.5–10.

SRHR, sexual and reproductive health and right.

Virtual semistructured interviews were held with 16 individuals from WHO, MoH and Vital Strategies between September and November 2022. All 16 participants were introduced to the evaluation and made aware of the ethical guidelines of the United Nations Evaluation Group^15^ being applied to the study and were assured of their confidentiality and the independence of the evaluator. The interviews lasted up to 60 min and followed a topic guide developed based on the desk review and the theory of change. The topic guide included questions about participants’ experiences of the workshop and policy dialogue implementation, the process of policy dialogue and results from its implementation, and the contribution of the initiative to any policy changes. Participants were also asked to reflect on lessons learnt from the experience and recommendations for future work. Extensive notes were taken, but interviews were not recorded. Follow-up interviews were conducted to gather additional details in February 2024.

### Patient and public involvement

As this paper does not directly concern service delivery, no patient involvement was included in this programme evaluation. However, incorporating public input—potentially including women’s groups and healthcare workers—could be beneficial in future work aimed at addressing some of the gaps and challenges highlighted in this paper.

## Results

### Experiences of the initiative

During the interviews, participants reported positive experiences of the initiative, with many highlighting that they appreciated the learning environment and the opportunity to understand the experiences of other countries. Participants felt the initiative responded to priority SRHR issues as representatives in each country selected their policy area based on their national agenda. Combining coaching with practical technical support for the implementation of policy dialogue activities allowed participants to put learning into practice. Participants particularly valued the hands-on practical approach to learning and appreciated the role-play elements in which they learnt how to present policy issues to policymakers and the media. In the postworkshop feedback surveys, participants also reported they appreciated learning from peers with different roles in different countries.

Participants also noted some challenges with the initiative. Drafting policy briefs before the in-person workshop was difficult for some participants who felt they lacked the skills to complete these preparation activities. However, support from coaches helped to address this challenge. Although the initiative facilitated learning across countries, language barriers between francophone and anglophone participants obstructed cross-country learning in some cases. Only two of the six country offices requested support from Vital Strategies in the third stage of the initiative, which might be because country teams received extensive support and coaching from WHO regional office technical officers instead. Despite the focus on training and capacity building in the initiative, participants felt that even more training would have been beneficial.

In postworkshop surveys, almost all participants reported that the workshop had increased their ability ‘moderately’ or ‘a lot’ (on a four-point scale) to identify policy options (94%), to adapt communication messages for different stakeholders (97%) and to communicate with media about a policy change (97%). After the workshops, almost all participants felt more confident than before to complete stakeholder analysis (97%), to draft a persuasive policy brief (100%) and to draft an action plan for policy change (97%). In the interviews, all participants reported enhanced knowledge and practical skills for policy engagement as a result of participation in the initiative. Specifically, participants highlighted they had learnt about how to work with SRHR policy opposition, and they reported increased awareness of the needs, priorities and motives of policy-makers, recognising their roles as bureaucrats rather than technical specialists.

### Quality assessments of the policy briefs

The evaluator reviewed six policy briefs to assess their quality and effectiveness in informing and supporting policy processes against this template. The evaluation revealed that while the identification of SRHR problems was consistently strong, the exploration of alternative solutions was notably weak (table 3). This limitation could hinder the ability of policy-makers to fully understand the range of potential interventions and their implications. Half of the reviewed briefs were deemed sufficient in their overall quality, while the other half required additional details to be fully effective. Stakeholder analyses were also reviewed by the independent evaluator. For the stakeholder analyses, the criterion ‘clearly written’ scored highest while the criterion ‘identification of stakeholder contributions’ received lower scores in the two countries. Although the stakeholder analyses included strategies for engaging each stakeholder, more detailed strategies were needed.

### Policy progress and factors influencing policy change

The policy progress reported by participants is outlined in table 2. Participants reported the initiative had contributed to policy change by motivating the teams to feel that policy change was feasible and by encouraging teams to be systematic and consistent in pursuing policy goals. The motivational effect of the initiative was facilitated by the requirement for participants to share plans and progress among countries. Participants also felt that the initiative strengthened the effectiveness of their policy dialogue activities. For example, the workshop supported participants to practise communication skills and ensure messages were delivered to policy officials succinctly. Having detailed feedback from mentors on supporting documents improved their quality. Participants highlighted that the stakeholder analysis was particularly useful for increasing the effectiveness of policy dialogue activities, as this comprehensive exercise encouraged the teams to mobilise all relevant stakeholders while clarifying in advance how key stakeholders needed to be engaged and how opposition could be overcome. Participants also felt the policy brief facilitated achieving policy change because it offered a clear framework for ensuing discussions with stakeholders and encouraged teams to have a tangible ask. Additionally, the process of developing the policy brief ensured there was a clear consensus on the priority policy area.

Based on the reports of country teams, there was progress with SRHR policy implementation in most participating countries evaluated (table 2). Elaborated below, participants reported several enabling factors that influenced their progress, including political context, specificity of the policy goal, alignment with government and donor priorities, and partnerships. Other factors that facilitated policy progress were the strong collaboration between WHO and MoH representatives, the wide range of knowledge and skills in national teams, and the inclusion of high-level leadership from WHO which increased access to policymakers and lent credibility to policy dialogue activities.

Political context: Effectiveness in influencing policy change varied depending on the political context in which policy engagement occurred. For example, the implementation of maternal and perinatal death surveillance and response (MPDSR) systems in Pakistan was slowed by the realisation that a bill would need to be passed through parliament to make MPDSR systems obligatory and that separate bills would be required in each devolved province. Although provincial-level bills have been drafted and there have been repeated consultative meetings in each province since the evaluation, the process of passing bills through parliament takes time. The requirement to work with provincial as well as national level governments was also a challenge in Nepal. The Nepal team addressed this by working closely with the provincial Health Directorates and other stakeholders to prioritise SRHR services. The Nepal team was particularly successful in having their policy for decentralised certification of abortion facilities accepted at the federal level, and the policy is now being implemented with government funding.^16^

Specificity of policy goals: Successful policy changes partially relied on the specificity of the policy goal. For example, the India team decided to focus on a specific policy aspect of a high-profile programme, which resulted in an early policy win. Two reproductive health indicators (adolescent birth rate and demand satisfied by modern methods of contraception) were rapidly included in the Transformation of Aspirational Districts Programme by November 2019. Participants reported this was facilitated by the country team’s coordination with high-level officials as well as the very clear and specific policy ask. Countries that chose broader and more ambitious goals relating to UHC saw slower progress, and goals had to be narrowed in some cases. Before the policy dialogue, Benin had piloted a health benefit package covered by national health insurance. The pilot evaluation followed by the policy dialogue led to recommendations to include abortion and other sexual and reproductive health services into the health benefits package. Burkina Faso initially intended to integrate comprehensive SRHR into primary care, but due to changes in staffing at both WHO and the MoH, the goal was narrowed to become more achievable, with the team instead focusing on community-level provision of family planning.

Alignment with government and donor priorities: Policy progress was facilitated by aligning selected policy goals with existing health system strengthening priorities and donor and government funding. In India, the policy goal was framed in terms of achieving the Sustainable Development Goals, which helped to align the policy with government priorities. However, in Rwanda, participants reported that their goal to increase financial resources for SRHR and to include SRHR in the essential package of health services was not met because this did not align with the government’s focus on non-communicable diseases and community health insurance. In this case, it was important for the team to be flexible and identify other policy goals that could be achieved and were in line with the government’s priorities. Instead, the team was successful in pursuing the national adoption of WHO guidelines for abortion care, family planning, and adolescent SRH, and revision of the national essential medicines list to include the combination pack of mifepristone and misoprostol as well as each medicine separately. Alignment with donor funding also enabled policy progress. The policy dialogue initiative was part of a larger SRHR project that involved collaboration between the WHO and MoH in each country, so relationships and commitment to SRHR had already been strengthened by dedicated resources in each country. Similarly in Pakistan, the policy work followed on from an existing MPDSR project that had provided valuable technical information to support evidence-based advocacy. Following the policy dialogue initiative, the team were able to apply newly developed policy skills to communicate technical information to relevant stakeholders.

Partnerships: Partnerships were critical for the policy dialogues, including with other United Nations (UN) agencies, civil society stakeholders and key government agencies at national and subnational levels, like the Ministries of Finance and Justice. In Nepal and Pakistan, partnerships with UNFPA enabled policy implementation to be divided by province or by level of the health system. This facilitated the nationwide implementation of facility certification in Nepal and supported preparation for implementation of compulsory MPDSR systems in Pakistan. Working with non-health government departments was also key. In Rwanda, WHO closely engaged the Ministries of Finance and Treasury departments to address health financing issues. The stakeholder analyses were, therefore, particularly important to facilitate effective partner engagement. For example, in India, the stakeholder analysis helped the team clarify who the ultimate decision maker for their policy ask would be, and therefore, what role the MoH should play in leading the policy dialogue.

Participants also reported factors that constrained the impact of policy dialogues: competing priorities limited the time available for staff to pursue policy dialogue activities, the COVID-19 pandemic moved attention away from non-COVID-19-related issues, and lockdown measures limited in-person stakeholder meetings. Additionally, linkages between WHO and MoH representatives were not as strong as required in all countries.

### Learnings for SRHR policy dialogues

There were several SRHR-specific issues that influenced the effectiveness of policy dialogues. Overall, participants reported that a higher level of engagement, evidence and hands-on implementation was sometimes needed for SRHR policy dialogues compared with other areas of health. For example, misconceptions among government officials about the safety of abortion care or the level of medical care required for family planning services posed challenges in some countries. In Nepal, there were concerns among national and provincial government stakeholders that provincial governments would not be able to ensure the quality of abortion care if certification of abortion facilities were decentralised. This was despite certification not being required for any other health service and despite abortion being a simple healthcare intervention that can be effectively managed by a wide range of health workers. The team held meetings with stakeholders to understand their concerns and then hired consultants to build the capacity of provincial governments to quality assure facilities for abortion care. This enabled the decentralisation of the certification process. Similarly, in Burkina Faso, there was concern among some stakeholders about the safety of family planning being provided at a community level, across all levels of health facilities. This concern was partly addressed by arranging a study visit to Madagascar for MoH officials to see how the proposed policy was being implemented elsewhere. Having strong evidence was also key to addressing concerns or misconceptions about SRHR, so the process of developing an evidence-informed policy brief was useful for policy dialogues. However, some countries needed to generate new local evidence to support the policy goal: for example, Nepal collected evidence to show the need for abortion facility certification to be decentralised. In Burkina Faso, the team supported a pilot of the proposed policy, collected evidence of safety and feasibility, and then engaged partner organisations to facilitate broader implementation beyond pilot districts.

As SRHR is often politicised, teams found it was also important to bring people on board incrementally, ensuring strong support and ownership from within the government to avoid backlash. This was facilitated by the initiative, which encouraged strategic and comprehensive stakeholder engagement and included training on handling opposition. The initiative’s focus on working with opponents was an important facilitator for policy change in Nepal: civil society organisations were initially opposed to the policy idea, but the inclusion of these organisations in quarterly meetings resulted in them becoming proponents for change. Participants in India also highlighted the need to be prepared for SRHR policy work to become politicised and to be exact and specific when advocating for new SRHR indicators, to ensure that rights-based indicators are implemented. In Pakistan, the SRHR intervention being proposed was not very sensitive due to its focus on addressing the dual burden of maternal and perinatal health, which can be less stigmatised than other areas of SRHR such as abortion or family planning. However, the MPDSR does include reporting on deaths from unsafe abortion, which will enable future work on addressing the quality of postabortion care and other causes of unsafe abortion. By addressing unsafe abortion within the wider umbrella of maternal health, it was possible to make progress without the work becoming contentious.

The skills developed through this policy dialogue intervention have since been applied by country teams to other SRHR policy goals. The WHO team in India applied their learnings to support amendments to the Medical Termination of Pregnancy Act, which raised the gestational limit for abortion in 2021. To manage sensitivities around this process, the team used specific learnings from the training, such as the need to frame the policy ask in terms of wider government priorities, to build consensus among a range of stakeholders before approaching government, to be prepared to take advantage of moments of opportunity and to carefully manage opposition. Similarly, the Pakistan team has since advocated for abortion to be included in the essential package of health services. This also involved careful management of opposition, and the team found that various skills from the workshop helped to achieve this goal. For example, producing a strong summary of the evidence, preparing the arguments and counterarguments that stakeholders might make in advance, and ensuring that plenty of supportive stakeholders attended relevant meetings. The team also found that the stronger working relationships developed with MoH officials throughout the initiative enabled their advocacy efforts.

## Discussion

There were several limitations associated with the evaluation findings. This evaluation design was participatory; subsequently, those who had designed and implemented the initiative were interviewed alongside participants. While they were assured of anonymity, their accounts of the intervention and its impacts may have been subject to courtesy bias if they tended to over-report positive feedback. Furthermore, participants were interviewed 3 years after the initiative started, and their reports may be subject to recall bias. The initiative’s effect on knowledge and competency was based also on participant self-report, rather than an objective test, though the systematic external quality assessment of the policy briefs and stakeholder analyses addressed some of these limitations. The policy changes cannot be directly attributed to the policy dialogues or the initiative as policy change is subject to multiple complex factors related to timing, people and context.^3^ Finally, it is possible that the findings cannot be generalised to all countries with an interest in SRHR policy dialogue. The participating countries had dedicated donor-funded SRHR programmes of work that focused on SRHR. This may have increased the likelihood of successful policy dialogue and adoption due to the existence of established relationships between WHO and the MoH in each country and increased engagement from senior representatives. Only 6 of the 15 countries involved in the policy dialogue were selected for the evaluation, and the results may not be applicable to all fifteen countries.

Despite these limitations, the implementation and evaluation of this initiative demonstrate the value of capacity building and ongoing support for policy dialogue for progressing SRHR policy change. We recommend further investment in strengthening the technical skills required for this approach to address SRHR policy gaps in future work. We also identified several important lessons for improving policy dialogue skills. We identified a need for better coordinated postworkshop support that more closely meets the needs of participants and increased preworkshop support for participants who felt they lacked the skills to complete policy briefs and stakeholder analyses in advance. Additionally, teams were encouraged to select policies that could be achieved in a short time frame, as the initiative only lasted 6 months, but policy progress takes time, and longer-term support is likely required. However, the combination of coaching with an in-person workshop worked well, and the inclusion of a coaching phase for any such initiative is essential. Participants found it particularly valuable to learn from other countries, but more language support is required in the future to maximise the potential benefits of cross-country learning. The policy brief and stakeholder analysis quality assessments suggest the need for coaches and participants to have greater focus on presenting a range of proposed solutions rather than one single solution to policy problems and identifying individual strategies for stakeholder engagement. There was also a need for more focus on timeframes and the level of investment required for policy dialogues. Although the initiative encouraged country offices to select policy goals with a short time span, some countries struggled to get longer-term, broader policies implemented, and staff turnover and resource shortages also created challenges for some.

We have also identified the additional considerations and requirements that may be needed for SRHR policy dialogue, compared with other areas of health, which can enable greater integration of SRHR into broader health policy dialogues in the future. Misconceptions about the level of medical infrastructure required for SRHR services and the potential for SRHR initiatives to be politicised meant that a higher level of engagement, evidence and implementation support was required in some countries to achieve policy change. Policy dialogue activities need to be well resourced.^2 5 12^ Our experiences suggest that the resource requirements for policy dialogue activities in SRHR can be heightened further. The need for resources was also greater when the subnational government needed to be engaged. We identified other factors that facilitated country teams to make policy progress despite the politicised nature of SRHR. These included working incrementally to ensure strong ownership within government, building strong support networks, careful management of opposition, preparation for backlash or unintended consequences and preparedness to seize moments of opportunity. Working with a broad range of stakeholders across national and subnational government and civil society, including non-health government departments, was therefore important. Close collaboration with MoH and other relevant agencies can also enable a strong understanding of national and subnational legal and political systems and can provide clarity on existing government priorities. This can facilitate the selection of a targeted and relevant policy goal which is valuable because engaging stakeholders on issues in which they have greater interest or investment can be a critical success factor for policy dialogue.^12^ Inclusion of more sensitive SRH topics within less sensitive ones, or framing SRHR initiatives within broader government goals (such as goals for female empowerment or sustainable development) can also enable progress.

## Data Availability

No data are available.
